# Novel oxygen-generation from electrospun nanofibrous scaffolds with anticancer properties: synthesis of PMMA-conjugate PVP–H_2_O_2_ nanofibers, characterization, and *in vitro* bio-evaluation tests†

**DOI:** 10.1039/d1ra02575a

**Published:** 2021-06-04

**Authors:** Samar A. Salim, Elbadawy A. Kamoun, Stephen Evans, Tarek H. Taha, Esmail M. El-Fakharany, Mohamed M. Elmazar, A. F. Abdel-Aziz, R. H. Abou-Saleh, Taher A. Salaheldin

**Affiliations:** Nanotechnology Research Center (NTRC), The British University in Egypt (BUE) El-Sherouk City Cairo 11837 Egypt e-b.kamoun@tu-bs.de badawykamoun@yahoo.com; Biochemistry Group, Dep. of Chemistry, Faculty of Science, Mansoura University Egypt; Polymeric Materials Research Dep., Advanced Technology and New Materials Research Institute (ATNMRI), City of Scientific Research and Technological Applications (SRTA-City) New Borg Al-Arab City 21934 Alexandria Egypt; Molecular and Nanoscale Physics Group, School of Physics and Astronomy, University of Leeds LS2 9JT UK; Environmental Biotechnology Dep., GEBRI, City of Scientific Research and Technological Applications (SRTA-City) New Borg Al-Arab City 21934 Alexandria Egypt; Protein Research Dep., GEBRI, City of Scientific Research and Technological Applications (SRTA-City) New Borg Al-Arab City 21934 Alexandria Egypt; Faculty of Pharmacy, The British University in Egypt (BUE) El-Sherouk City Cairo 11837 Egypt; Biophysics Group, Dep. of Physics, Faculty of Science, Mansoura University Egypt; Nanoscience and Technology Program, Faculty of Advanced Basic Science, Galala University Egypt; Pharmaceutical Research Institute, Albany College of Pharmacy and Health Sciences Abany NY 12144 USA Taher.salaheldin@acphs.edu

## Abstract

Released oxygen plays a critical role in reducing destructive tumor behavior. This study aims to utilize decomposed hydrogen peroxide as an oxygen source by conjugating it with polyvinylpyrrolidone (PVP). PVP–hydrogen peroxide complex (PHP) composed of different ratios of (PVP : H_2_O_2_) (0.5 : 1, 1 : 1, 1 : 1.5, 1 : 5, and 1 : 10) were successfully synthesized. PHP complex with a ratio of 1 : 1.5 was chosen as the optimized ratio, and it was incorporated into the polymethyl methacrylate (PMMA) nanofibrous scaffold *via* the electrospinning technique. Results have revealed that the PMMA–10% PHP complex provided a significant morphological structure of nanofibrous scaffolds. The mechanical properties of PMMA–10% PHP nanofibers showed the most suitable mechanical features such as Young's modulus, elongation-at-break (%), and maximum strength, in addition to the highest degree of swelling. All PHP complex scaffolds released oxygen in a sustained manner. However, the PMMA–10% PHP complex gave the highest concentration of released-oxygen with (∼8.9 mg L^−1^, after 2.5 h). PMMA–10% PHP nanofibers provided an ideal model for released-oxygen scaffold with anti-cancer effect and high selectivity for cancer cells, especially for breast cancer cells. Nanofibrous scaffolds with different composition revealed high cell viability for normal cells. Such outcomes support the suitability of using synthesized nanofibrous scaffolds as released-oxygen biomaterials to enhance cancer cells' sensitivity and maximize the treatment effect.

## Abstract 2

Released oxygen plays a critical role in reducing destructive tumor behavior.

## Graphical abstract

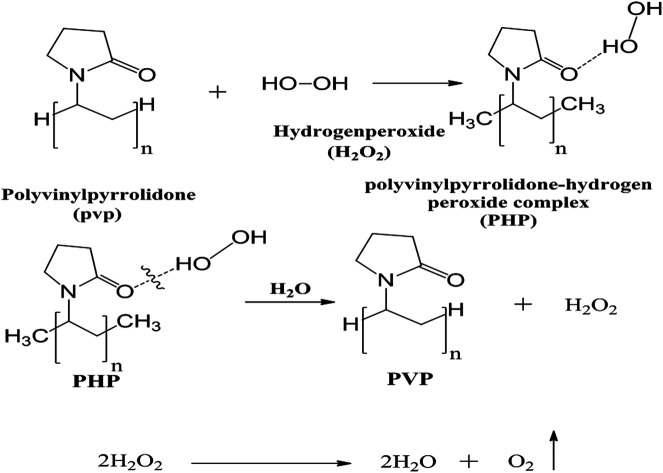

## 1 Introduction

Most solid tumors develop due to hypoxia, when the normal cells suffering from an insufficient oxygen supply are converted to cancer cells. Recent studies have demonstrated that tumor hypoxia is a critical obstacle for effective cancer treatment with chemotherapy, immunotherapy, as well as radiotherapy.^1^ Patients exposed to hyperbaric oxygen (HBO) immediately before irradiation show a significant improvement in the efficacy of radiation therapy. Oxygen has also been shown to increase the cyto-static effect of chemotherapy treatment.^2^ Therefore, various sources of oxygen are being explored, such as oxygen-releasing biomaterials, for *e.g.*, solid inorganic peroxide such as sodium percarbonate,^3^ calcium peroxide,^4,5^ magnesium peroxide,^3^ and liquid peroxide as hydrogen peroxide^6,7^ in the attempt to reverse the occurrence of hypoxia. Further, multiple studies have revealed that the hypoxic environment of the tumor has a critical role in regulating cancer metastases, specifically *via* the hypoxia-inducible factor 1 (HIF-1), which occupies a vital role in controlling the hypoxic response.^8^ Oxygen enhanced the degradation of HIF-1 and thus locked genes, which are activated by the hypoxic environment.^9^ Hypoxia is considered to be the main factor for stimulating the transition of epithelial cells (cobble-stone shape) to mesenchymal cells (flat-spindle shape), with high potential for invasion, motility proteins, and metastatic niche formation.^10^ This biological transition process is called the epithelial–mesenchymal transition (EMT). Accordingly, there is a strong relationship between tumor progression and hypoxia.

Recently, the fabrication of nanostructured materials, particularly in the form of nanofiber mats, has gained interest for versatile applications. The nanofiber structure has many advantages, such as increased surface area, controlling the release rate profile, and increased fiber strength by decreasing defects on the fiber surface. As a result of these unparalleled properties, nanofibers could be utilized in different biomedical purposes, *e.g.*, drug delivery,^11^ tissue engineering,^12^ and wound dressings.^13^ The fabrication of nanofiber scaffolds using the electrospinning technique offers potential due to its flexibility and cost-effectiveness and has gained significant attention for biomedical applications.^14^ Thus, nanofibrous scaffolds are suitable to promote cell proliferation and cell adhesion for wound dressing;^15–18^ in addition, they have also been used for assisting tissue regeneration. Poly(methyl methacrylate) (PMMA) has been widely used as a synthetic polymer for biomedical purposes due to its mechanical properties, its good biodegradability and biocompatibility, non-toxicity, dimensional stability, and has no taste or odor.^19^ Furthermore, cell adhesion and stability in the body fluid represent important properties of PMMA.^20,21^ Currently, PMMA is mostly used in dentistry as a promising bio-compatible material,^22^ as bone cement in orthopedic surgery,^23^ as well as in intraocular lenses.^24^

Electrospinning is an extensively used technique for the electrostatic production of nanofibers; it fabricates nanofibers with diameters ranging from ∼2 nm to several micrometers based on polymer solutions or melts. This process has the ability to continuously produce nanofibers on the scale of nanometers, which is difficult to achieve using other techniques. The key principle of the electrospinning process is applying high voltage on a polymer solution, which induces a drop of the polymer solution to be stretched into nanofibers. There are several parameters that can expressively affect the creation and morphology of the produced nanofibers. These parameters are commonly divided into three groups: solution parameters (*e.g.*, viscosity, polymer concentration, molecular weight of polymer, solvent type, surface tension, and conductivity), and spinning process parameters (*e.g.*, voltage applied, collecting distance, and polymer feeding rate), as well as atmospheric parameters (*e.g.*, temperature and relative humidity of room spinning).^25^

Lately, numerous review articles have provided an overview of the electrospinning technique for a number of applications. Torres *et al.*, studied the electrospun nanostructures as advanced bioactive scaffolds for tissue engineering, food packaging, drug delivery, as well as functional coatings.^26^ The electrospinning of bioactive polymers was well reviewed including naturally polymers, synthetic polymers, in addition to polymer blends, and also the functionalization of the electrospun surfaces by combination of bioactive substances. Wen *et al.*, discussed the incorporation of bioactive compounds in electrospun nanofibers for food applications.^27^ Coaxial electrospinning and the used natural biopolymers were summarized by Zhang *et al.*, which offer new strategies to develop novel functional foods.^28^

Hydrogen peroxide decomposes into oxygen and water in the presence and absence of a catalyst or enzyme such as catalase, which is present in many cells in the human body, especially liver and blood.^29^ Hydrogen peroxide has been used widely in biomedical applications due to its ability to kill microorganisms such as bacteria, fungi, and protists, even at low concentration,^30^ and has thus been used extensively in oral health-care products.^31^ Hydrogen peroxide could be loaded onto polyvinyl pyrrolidone (PVP) to create the PHP complex (PVP–hydrogen peroxide).^32^

Recently, calcium peroxide (CaO_2_)-loaded high concentration up to 10% into poly(glycerol sebacate) (PGS) and poly(ε-caprolactone) (PCL) scaffolds was used as the source of oxygen.^33^ This composite scaffold demonstrated the sustained release of oxygen for many days and significantly enhanced the cell metabolic activity owing to the reduction of the hypoxic environment around the bone-marrow-derived mesenchymal stem cells (BM-MSCs). Furthermore, the scaffolds also indicated good antibacterial performance. However, the use of CaO_2_ as a source of oxygen is required to be prepared as nanoparticles to guarantee good dispersion and homogeneity when loaded on the nanofibrous scaffold.

Herein, H_2_O_2_ was used as a source of oxygen in the form of the PHP complex loaded-nanofiber scaffolds for the first time in literature. The toxicity associated with a high dose of released oxygen from hydrogen peroxide decomposition was avoided by loading H_2_O_2_ onto PMMA nanofibers, offering the sustained released oxygen. In particular, we provided the first demonstration of the PMMA nanofibrous scaffold loaded-PHP complex as a source of oxygen. We demonstrate the anticancer effect of the synthesized PHP complex, even in low concentration, and furthermore show that the released oxygen was controlled and provides a sustained release profile. The physicochemical properties of the PHP-loaded PMMA electrospun nanofibrous scaffolds were determined in addition to assessing their *in vitro* biocompatibility, cytotoxicity, anticancer properties, and finally their potential as a novel oxygen-releasing biomaterial for cancer treatment.

## 2 Materials and methods

### 2.1. Materials

Poly(methyl methacrylate) (PMMA, *M*_w_ 550 000 g mol^−1^) and poly vinylpyrrolidone (PVP, *M*_w_ 40 000 g mol^−1^) were obtained from Alfa-Aesar, Germany. Hydrogen peroxide ∼30% and acetone 99% (extra pure) were obtained from Biochem, UK. An electrospinner device (MECC, NANON-01A, Japan) was used for nanofiber fabrication.

### 2.2. Preparation of the PHP complex with different ratios of (PVP : H_2_O_2_)

**Fig. 1:**
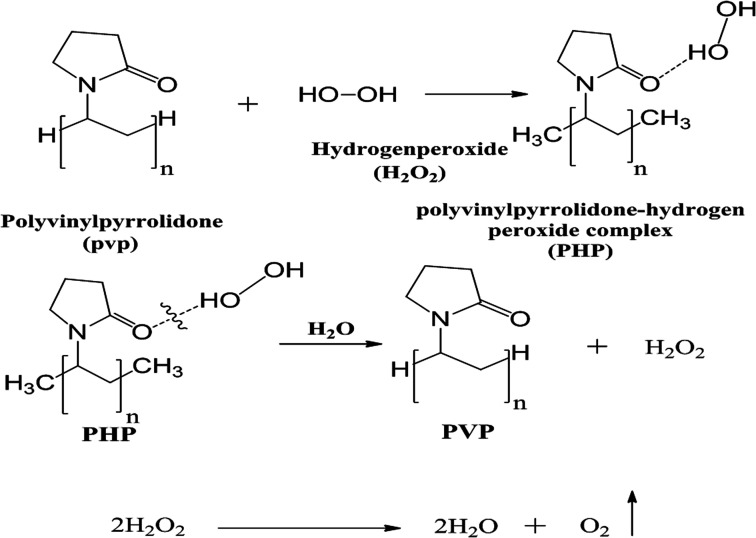
Scheme of PHP preparation and oxygen release.

A distinct weight of PVP was added to the corresponding volume of pre-cooked H_2_O_2_ (30%, w/v) in a glass beaker according to different ratio (PVP : H_2_O_2_) as (0.5 : 1, 1 : 1, 1 : 1.5, 1 : 5, and 1 : 10); then, the solution was kept under continuous stirring at 250 rpm for 2 h. The resultant solution was poured in a specific bottle of a freeze dryer for lyophilizing and kept overnight at (0.001 mbar at −80 °C) (Fig. 1). Ultimately, a solid powder was attained after drying and pressed using a mortar. The resultant PHP crystals were stored at 2–8 °C for further use. The solid PHP complex was created as the oxygen source according to the previously developed method by Modhave *et al.*, with slight modifications.^32^ In addition, the yield of the PHP complex is calculated theoretically.

### 2.3. Treating of PVP using lyophilizer

A known weight of PVP (6 g, ∼65%, w/v) was dissolved in 9 mL of de-ionized water. Afterwards, the PVP solution was kept under continuous stirring at 250 rpm for 2 h. The PVP solution was moved to specific bottles of the freeze-dryer for lyophilizing and kept overnight at (0.001 mbar at −80 °C). Afterwards, the solid powder was crushed using mortar and the obtained PVP solid powder was stored at 2–8 °C for further experiments.

### 2.4. Fabrication of the PMMA/PHP complex nanofiber scaffolds

PMMA (0.6 g, *i.e.*, 6%, w/v) was dissolved in acetone under continuous stirring overnight at room temperature in a tightly closed vial. The PHP complex was added to the PMMA solution in different ratios (5, 10, and 15%). The blend ratios of (PMMA/PHP) were mixed and kept under stirring overnight or till reaching a homogenous one-phase solution in a tightly closed vial, followed by moving to an ultrasonicator for 10 min at room temperature before the spinning step. Different scaffolds composed of the PMMA/PHP nanofibrous mats were fabricated by an electrospinner. The polymer solution was collected in 6 mL plastic syringe joined with a Teflon tube (PTFE tube) and a stainless-steel needle diameter 22 G fixed at the end with a metal connector, all of these mounted in the electrospinner system and subjected to high voltage up to 27 kV, and RH ∼ 55%.

### 2.5. Characterization of the PMMA/PHP nanofibrous complex scaffolds

#### 2.5.1. FTIR

IR (IR, 8400s Shimadzu, Japan) finger-prints in the transmittance mode were recorded in the range of 4000–400 cm^−1^ to investigate the chemical composition of the PMMA/PHP scaffolds.

#### 2.5.2. SEM

The nanofiber surface morphology of different scaffold compositions was examined by a scanning electron microscope (ESEM, Quattro S, Thermo Scientific, USA) instrument with an acceleration voltage of 5–30 kV. Well-dried samples were carefully sectioned into appropriate size, then fixed on a specific grid. Nanofiber samples were coated by one cycle thin layer of Au before investigation using Desk Sputter Coater (Vac-Techniche, UK).

#### 2.5.3. Dissolved oxygen (DO) measurement

The concentration of DO released from the PMMA/PHP complex nanofibrous scaffolds with different composition ratios and PHP complex alone as a powder was measured by a DO probe (ORION VERSA STAR, Thermo-Scientific-Orion DO Probe, USA). Advanced electrochemistry meter was used to measure the concentrations of dissolved oxygen in a BOD bottle with volume 1 mL. Each scaffold was cut into identical pieces with almost the same size of about 20 μg and then immersed in one mL of deionized water. DO was measured for the PHP complex as a powder and liquid and submerged into one mL of deionized water. The DO concentrations were recorded at different time intervals. This procedure was coupled with the previously designated procedure of Voss *et al.*^34^ and Ahmed *et al.*^35^

#### 2.5.4. Mechanical strength

The tensile strength of different composition of scaffolds was determined by a standard uniaxial tensile test (Z050, Zwick Roell AG, Ulm, Germany). Specimen dimensions (40 × 10 mm) were stretched out at a speed of 10 mm min^−1^ with an initial length of 20 mm and 50 N of cell loads. Measurements were taken for three specimens of each composition to obtain average values and standard deviations.

### 2.6. Physicochemical properties of the PMMA/PHP nanofibrous complex scaffolds

#### 2.6.1. Swelling index (SI)

Appropriately sized scaffolds were weighed individually (*W*_d_) and after that, immersed in de-ionized water. After 10 min, swollen nanofibers were impassive and dried well by towel paper. Then, the swollen mats were re-weighed (*W*_s_) at different time intervals to detect the change in the sample weight, as given in eqn (1).

1 SI = (*W*_s_ − *W*_d_)/*W*_d_

#### 2.6.2. Hydrolytic degradation of nanofibrous complex scaffolds

To estimate the relative amount of weight loss (%) from different mats in an aqueous environment, each scaffold was cut in a significant format and weighed on a sensitive balance. After that, each piece was soaked in 15 mL of de-ionized water and kept in an incubator at room temperature for one of the following times: 0, 0.5, 1, 1.5, and 2 days. Subsequently, each piece was detached from water and weighed, then compared with the original dried weight. The loss of each mat was estimated depending on the weight loss for each scaffold.

### 2.7. *In vitro* bio-evaluation tests

#### 2.7.1. Antimicrobial activity of the PMMA/PHP nanofibrous complex scaffolds

##### Bacterial strains

The human pathogenic microorganisms (*Candida albicans* ATCC 700, *Escherichia coli* NCTC10418, *Klebsiella pneumoniae* ATCC13883, and *Bacillus cereus* ATCC6633) were kindly provided by the National Institute of Oceanography and Fisheries (NIOF), Alexandria, Egypt.

##### Disc diffusion assay

The disc diffusion assay was performed to test the ability of different nanofiber samples (PMMA, PMMA–5% PHP, PMMA–10% PHP, and PMMA–15% PHP) to stop the growth of bacterial and yeast pathogenic microorganisms. Overnight broth cultures of each microbial pathogen were prepared in 5 mL of Luria–Bertani broth and incubated at 30 °C using 150 rpm shaking condition. The grown microorganisms were diluted to 0.5 McFarland standard, followed by swab spreading over the surface of the nutrient agar plates. A single 0.7 mm disc of each nanofiber sample was aseptically and gently added to the plates' surfaces. The plates were incubated at 30 °C for 24 h, and then the formation of clear/inhibition zones were detected.^36^

#### 2.7.2. Hemocompatibility of the PMMA/PHP nanofibrous complex scaffolds

The testing of the ability of a compound or a chemical structure to burst the human RBCs and release their hemoglobin content is a considerable tool to investigate the biocompatibility of the tested nanofibers. In brief, a sample of healthy whole-blood was obtained and mixed with a few drops of EDTA solution to avoid its clotting. A total volume of 700 μL of Ca^2+^–Mg^2+^ free DPBS buffer were gently mixed with 10 μL of the collected blood. A total weight of 100 mg of samples (PMMA, PMMA–5% PHP, PMMA–10% PHP, and PMMA–15% PHP) were separately added to each tube of diluted blood. Moreover, 100 μL of Triton X-100 and DMSO (0.5%) were replaced in the samples in the positive and negative controls, respectively. Both the tested tubes and the control tubes were incubated at 37 °C for 3.5 h, followed by 30 min of interval inverting. All the tubes were subsequently centrifuged at 10 000 rpm for 15 min. A ratio of 1 : 1 of each sample and the cyanmethemoglobin reagent was prepared and the absorbance of each mixture was spectrophotometrically measured at 540 nm against the blank (Ca^2+^–Mg^2+^ free DPBS buffer and nanofibers without blood).^37^

#### 2.7.3. Cytotoxicity of the PMMA/PHP nanofibrous complex scaffolds

The effect of the samples on the cell viability of Vero normal cells (kidney epithelial cells of African green monkey) was investigated using the MTT cell viability assay, as previously described by Mosdam^38^ and Almahdy *et al.*^39^ In short, Vero (1.0 × 10^3^) cells were seeded in triplicate in 96-well sterile flat bottom tissue culture micro-plates and cultured in DMEM (Lonza, USA), supplemented with 10% fetal bovine serum (FBS), and then the cells were incubated at 37 °C in a 5% CO_2_ incubator for 24 h. Then, the nanofiber discs at concentrations of (250, 500, 750, 1000 μg mL^−1^) (Fig. 11b) and PHP at concentrations of (12.5, 50, 100, 150, 200, and 250 μg mL^−1^) (Fig. 11a) were added to the cells in triplicate and incubated at 37 °C in a 5% CO_2_ incubator. After incubation for 48 h, the nanofiber discs were removed and the cells were washed three times with 1.0 M PBS to remove the debris and dead cells. Then, 200 μL of the MTT solution (0.5 mg mL^−1^) was added to each well and incubated at 37 °C and 5% of CO_2_ for about 2–3 h. The formazan crystals were dissolved in 200 μL per well of DMSO and the absorbance was measured at 595 nm using a micro-plate ELISA reader. Cell viability (%) compared to the control wells containing the cells without adding nanofiber discs were calculated using the given formula: (*A*) test/(*A*) control × 100%.

The values of IC_50_ were determined using the Graphpad Instat 6.0 software. All the experiments were performed three times and untreated control cells (cells without adding any compound) were considered as the negative reference.

#### 2.7.4. Anticancer activity of the PMMA/PHP nanofibrous complex scaffolds

The antitumor activity of the tested samples was assayed *in vitro* by testing their cytotoxicity toward different tumor cell lines using the MTT method, as described above. Human cancer (Caco-2, MDA, and HepG-2) cells at concentrations of 1.0 × 10^4^ per well were seeded in 96-well flat-bottom plates overnight in 5% CO_2_ atmosphere. After cell attachment, the tested PHP complex at different concentrations 6.25, 12.5, 25, 37.5, 50, and 75 μg mL^−1^ and PMMA–PHP nanofibers discs at different concentrations (0, 250, 500, 750, and 1000 μg mL^−1^) were inserted into each well. All plates were incubated in the 5% CO_2_ incubator for 48 h. Then, the cells were washed three times with fresh medium to remove the debris and dead cells, and 200 μL of the 0.5 mg mL^−1^ MTT solution in PBS buffer were added to each well and incubated in the 5% CO_2_ incubator for 2–3 h to allow the viable cells to metabolize MTT. Then, cell viability and IC_50_ values were determined as described above and the selectivity index (SI) for each sample was estimated by dividing the mean IC_50_ of the Vero cells by the mean of IC_50_ of the tumor cells.

### 2.8. Statistical study

The data were analyzed using the analysis of variance (ANOVA) signed rank test at a significance level of 0.05. All the data are reported as mean ± standard deviation.

## 3 Results and discussion

### 3.1. Calculation of the yield (%) of the PHP complex

**Fig. 2:**
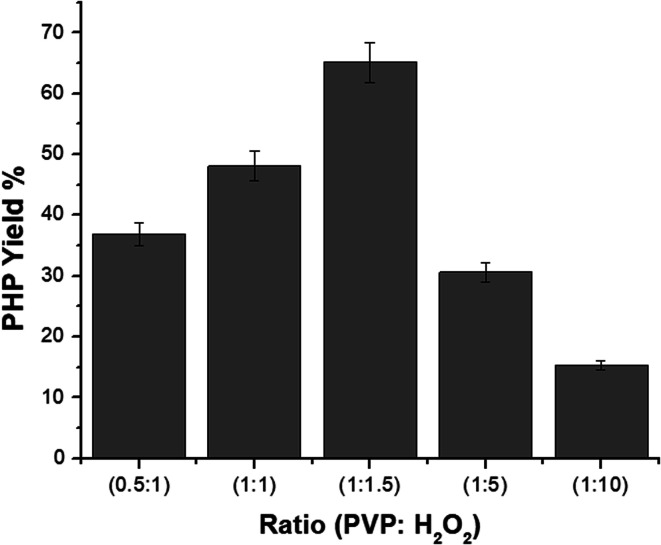
Representation of yield (%) of PHP complex with different ratios (1 : 10), (1 : 5), (1 : 1.5), (1 : 1) and (0.5 : 1) (mean ± sd, *n* = 3, *p* < 0.05).

The yield (%) of the PHP complex with different ratios after lyophilizing is shown in Fig. 2. The yield percentage was calculated as given in the equation.2
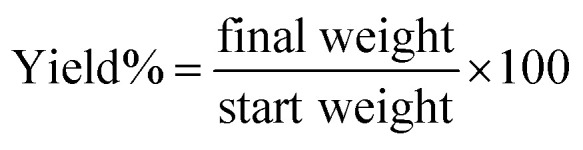


The different ratios of PVP : H_2_O_2_ used to obtain the complex were 0.5 : 1, 1 : 1, 1 : 1.5, 1 : 5, and 1 : 10, which provided a yield of 36, 48, 66, 30, and 15%, respectively. Notably, the yield (%) of the formed complex increased with increasing ratio of H_2_O_2_ till (1 : 1.5), after which it decreased. Accordingly, the ratio (1 : 1.5) of PVP : H_2_O_2_ gave the optimum yield (%) of the complex of 66%, compared to the yield (%) of other tested ratios.

### 3.2. Optimization of the spinning conditions

Previous studies have discussed the morphology of nanofibers by optimizing the parameters of the electrospinning process to improve the producible nanofibers without any droplets.^40^ Herein, different ratios of PMMA (6, 10, and 12%) were employed using various flow-rates, which were affected by the applied voltage and collected on different distances from the spinneret, as described in Table 1. The PHP complex was also added with different ratios (0, 5, 10, and 15%) to optimize the PMMA ratio. The optimum nanofibers from PMMA were obtained at a PMMA concentration at 6% by applying a high voltage of 27 kV and a solution feeding-rate of 4.5 mL h^−1^ at a distance 15 cm from the plate collector. All these scaffolds were fabricated using tubeless spinneret technique fixed with a needle with a diameter of 22 G and collected on a plate collector well covered with aluminum foil with a width of 40 mm. All the electrospinning experiments were carried out at the same ambient parameters as all of them were developed at room conditions with a RH of 40%. The scaffolds were made up under sterile condition. The fabricated scaffolds were dried at ambient conditions or in a dry oven at 40 °C to remove the residual solvent, and were then stored under dry/cold conditions to avoid any contamination (Table 1).

**Table 1:** Optimization of the spinning conditions of PMMA and PMMA–PHP nanofibrous scaffolds^a^

Sample	Voltage (kV)	Feed rate (mL h^−1^)	Distance between tip & collector (cm)	Fiber morphology observation
PMMA (6%)*	27	4.5	15	Beads-free nanofibers
PMMA (10%)	27	2, 3, and 4.5	15	Poor fibers with much beads
PMMA (12%)	27	2, 3, and 4.5	15	No nanofibers formed
**PMMA + 5% PHP complex	***27***	***4.5***	***15***	***Good nanofibers beads-less***
**PMMA + 10% PHP complex	***27***	***4.5***	***15***	***Beads free nanofibers***
**PMMA + 15% PHP complex	***27***	***4.5***	***15***	***Nanofibers with little beads***

a *: means optimum used condition, **: means a 6% PMMA. Cells in bold italic mean nanofibers with the PHP complex.

**Table 2:** IC_50_ (95% confidence interval) (μg mL^−1^) of PMMA nanofibrous scaffolds and the PHP complex with different ratios^a^

Sample	IC_50_ (95% confidence intervals) (μg mL^−1^) using different cell-lines			
	Vero cells	Caco-2 cells	MDA cells	HepG-2 cells
***PMMA + 0% PHP (control)***	***1836 (1422–2371)***	***1172 (1107–1241)***	***1267 (1123–1429)***	***837 (812.4–862.4)***
***PMMA + 5% PHP***	***2340 (1720–3182)***	***1003 (963.5–1045)***	***1103 (801.7–1518)***	***797.7 (780.3–815.4)***
***PMMA + 10% PHP***	***1670 (1361–2050)***	***671 (629.6–715.1)***	***644.8 (608.1–683.8)***	***717.6 (673.3–764.8)***
***PMMA + 15% PHP***	***2292 (2065–2544)***	***644.9 (581.8–714.9)***	***616.6 (568.9–668.2)***	***629.7 (589.3–672.9)***
PHP (0.5 : 1)	80.43 (75.57–85.6)	36.48 (32.7–41.21)	41.92 (40.15–43.78)	34.34 (31.08–37.95)
PHP (1 : 1)	48.62 (43.18–54.74)	25.29 (23.19–27.64)	37.93 (36.73–39.16)	31.14 (29.43–32.94)
PHP (1 : 1.5)	33.36 (28.98–38.41)	22.42 (21.17–23.75)	34.73 (33.26–36.26)	30.17 (27.99–32.51)
PHP (1 : 5)	38.81 (33.88–44.47)	21.2 (18.97–23.68)	32.21 (30.19–34.36)	32.05 (28.35–36.22)
PHP (1 : 10)	33.8 (29.63–38.57)	19.7 (17.92–21.66)	32.96 (31.73–34.23)	37.76 (28.28–50.43)
PVP (control)	987.5 (471.5–2068)	1037 (461.5–2328)	1795 (786.7–4093)	787.2 (392.8–1578)

a Cells in bold italic mean nanofibers with the PHP complex.

### 3.3. Characterization of the PHP complex and the PMMA nanofibrous scaffolds

#### 3.3.1. FTIR spectra of the PHP complex

**Fig. 3:**
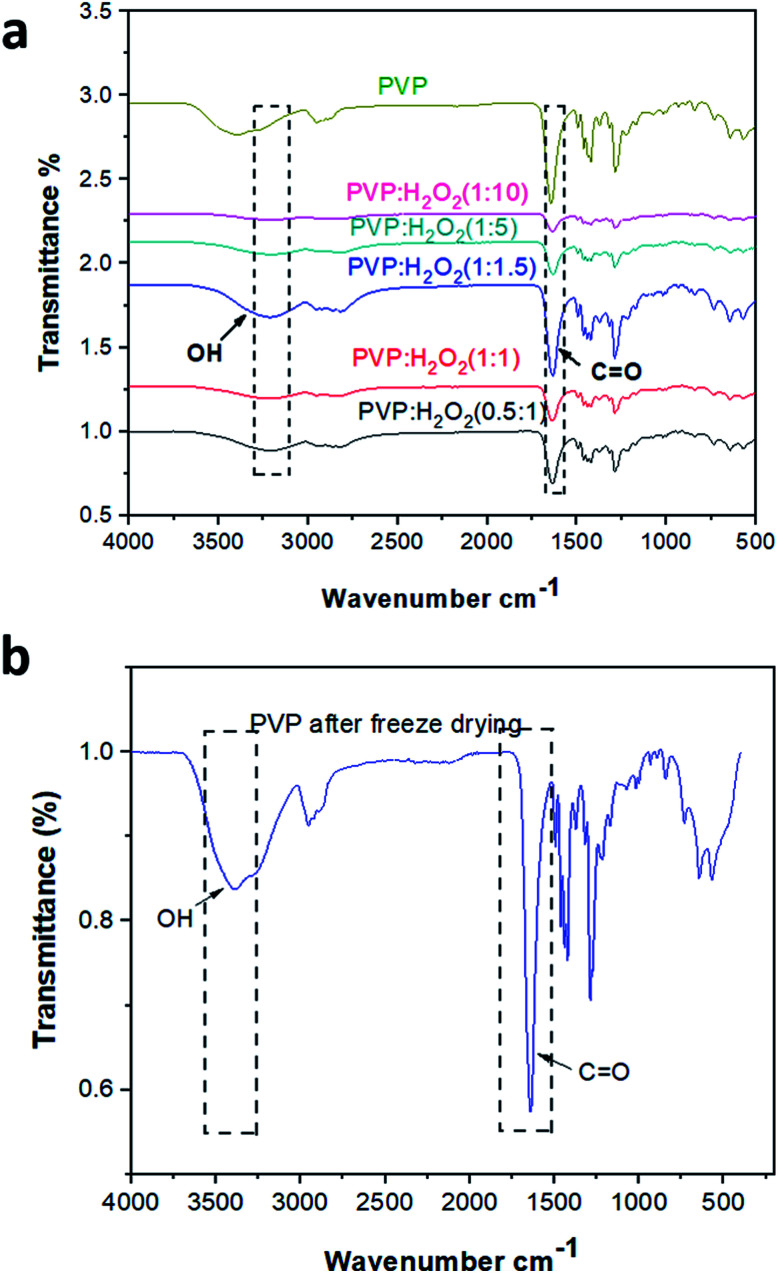
FTIR spectra of different ratios of PHP complex (a) and PVP (b).

Fig. 3 shows the FTIR spectra of the PHP complex produced by different ratios between PVP : H_2_O_2_ (0.5 : 1, 1 : 1, 1 : 1.5, 1 : 5, 1 : 10) and pristine PVP. It is obvious that the vibrational bands of PVP were detected at *ν* 1288, 1665, and 3400 cm^−1^, which are assigned to C–N, C=O, and O–H, respectively.^41^ On the other hand, the characteristic peaks of the PHP complex were assigned to the hydroxyl and carbonyl groups, which are obviously found in all the spectra of different ratios of the PHP complex, as represented in Fig. 3a. The formation of the hydrogen bond between PVP and H_2_O_2_ was proved by the shifting of the band of the carbonyl group from *ν* 1665 cm^−1^ to 1630 cm^−1^. Moreover, a vibrational broad band appeared at 3400 cm^−1^, pointing toward the presence of O–H at the end chains of PVP. In the case of the PHP complex, the visible distinct band appears at a maximum of *ν* 3100 cm^−1^, which suggests the presence of intermolecular hydrogen bonding between H_2_O_2_ and PVP. Notably, the characteristic peaks for the formation of the PHP complex (*i.e.*, –OH and C=O) were detected in all the tested ratios; however, these peaks seem to be clearer with the ratio of (1 : 1.5), as shown in Fig. 3a. Thus, this ratio was chosen as the optimum ratio for further experiments. This result was shown previously by Modhave *et al.*^32^ The PVP control was exposed to the same preparation conditions to confirm that the shifts are the result of PHP complex formation and not due to the process of lyophilization, which was further demonstrated in the IR spectra, as shown in Fig. 3b.

#### 3.3.2. FTIR spectra of the PMMA–PHP complex nanofibrous scaffolds

**Fig. 4:**
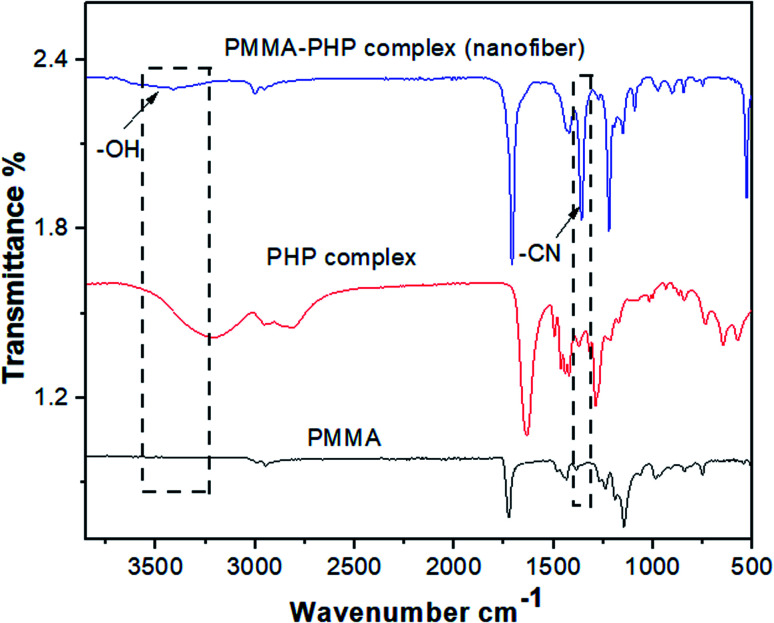
FTIR spectra of PMMA, PHP complex and PMMA–PHP nanofiber.

The IR spectrum of the PMMA–PHP complex shows the presence of a broad peak at *ν* 3100 cm^−1^ for the –OH group and a sharp intense peak at *ν* 1300 cm^−1^ owing to the C–N group, which is corresponding to the PHP complex (Fig. 4); these results are consistent with the results of Modhave *et al.*^32^ Furthermore, the vibrational peaks of PMMA clearly appear at *ν* 1200 cm^−1^ due to the presence of the ester bond C–O and a sharp vibrational band at *ν* 1700 cm^−1^ owing to the carbonyl group C=O, as previously reported by Vijayakumari *et al.*^42^ Moreover, the presence of common vibrational bands for both PMMA and the PHP complex at *ν* 2900 and 1700 cm^−1^ corresponding to CH_2_ and C=O groups, respectively (Fig. 4).

### 3.4. Morphology investigation of the nanofibrous scaffolds

**Fig. 5:**
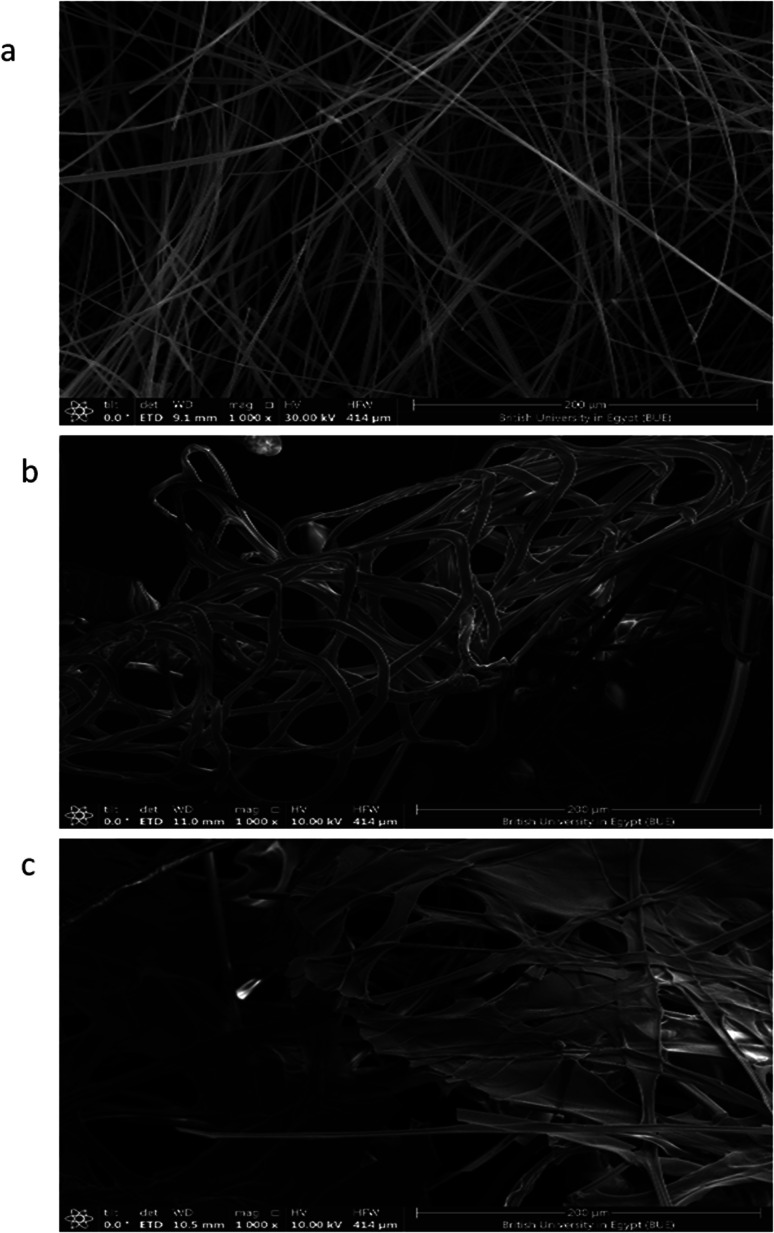
SEM photographs of PMMA nanofibrous mats of (a) 6% PMMA, (b) 10% PMMA, and (c) 12% PMMA.

The SEM images of the PMMA scaffolds with different concentrations of PMMA (6, 10, and 12%), are shown in Fig. 5. 6% PMMA displayed a high density of regular, continuous, and smooth nanofibers (Fig. 5a), compared to other concentrations of (10 and 12% PMMA), which provided poor and irregular nanofibers (Fig. 5b and c), respectively.

**Fig. 6:**
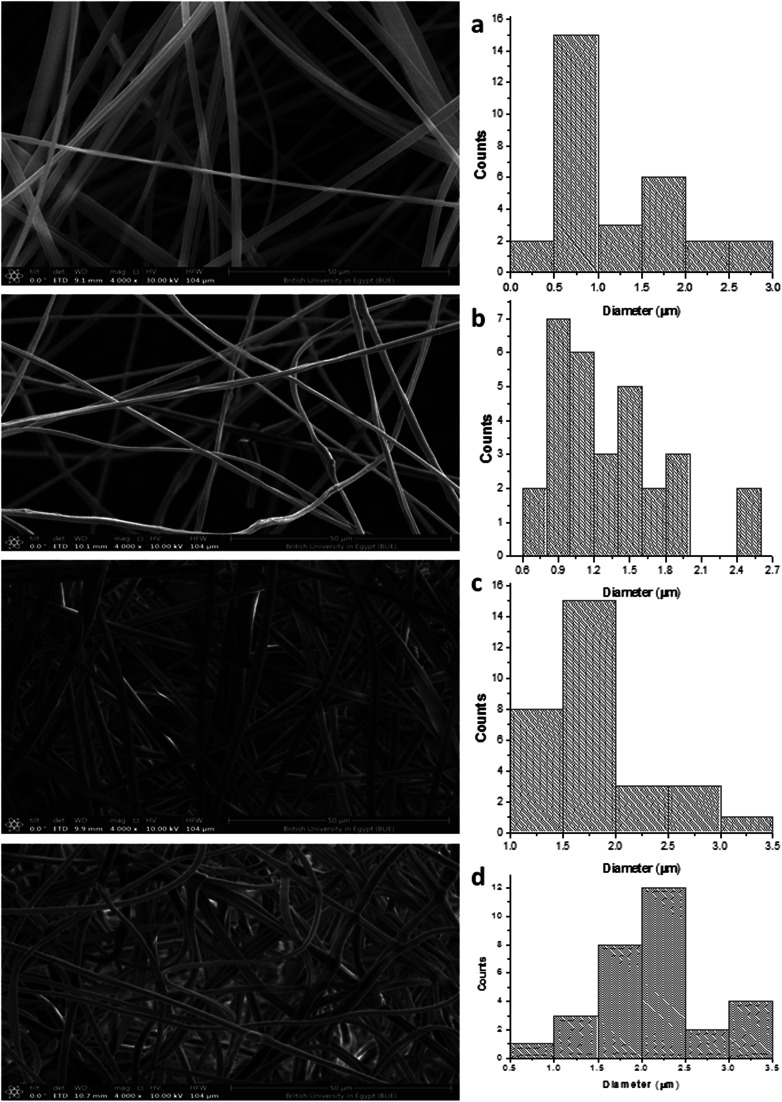
SEM photographs of PMMA nanofibrous mats of (a) 6% PMMA–0% PHP, (b) (6% PMMA–5% PHP), (c) (6% PMMA–10% PHP), and (d) (6% PMMA–15% PHP).

After the PHP complex loading of the 6% PMMA scaffolds with different concentrations (0, 5, 10, and 15% PHP), the fiber diameter was found to increase with loaded PHP (Fig. 6). Interestingly, the scaffold (6% PMMA without complex) gave a small diameter of nanofibers, about 0.7 μm, whereas the fiber diameters increased to (1.2, 1.7, and 2.3 μm) upon the loading of 5, 10, and 15% of the PHP complex (Fig. 6b–d), respectively. The increased nanofiber diameter is associated with the increased viscosity of the spinnable polymer solution, as previously reported by Sibokoza *et al.*^43^ They proved that the fiber's diameter of the PMMA NFs was increased by loading different concentrations of copper selenide (CuSe) with 1.4% PMMA NFs, which raised the size sharply from 1.4 μm to 10.1 μm, compared to PMMA NFs without CuSe. The SEM images might exhibit that the addition of high concentration of the PHP complex (15%) resulted in the formation of the sandwiched fiber phenomenon (Fig. 6d). However, the scaffold with 10% PHP complex showed the most regular and smooth nanofibers with high distribution and density, as clearly shown in Fig. 6c.

### 3.5. Swelling study of the PMMA–PHP nanofibers

**Fig. 7:**
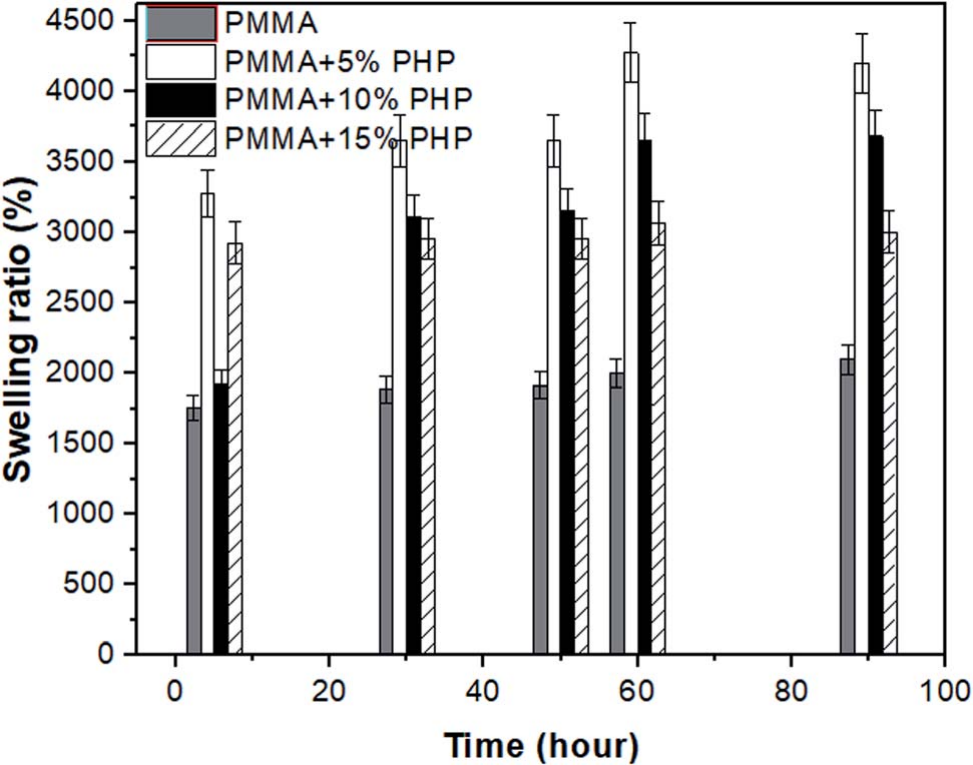
Swelling ratio of nanofibrous (PMMA–0% PHP, PMMA–5% PHP, PMMA–10% PHP, and PMMA–15% PHP) mats, (mean ± sd, *n* = 3, *p* < 0.05).

Nanofibrous scaffolds should absorb water or physiological fluids through their pores to facilitate cell signaling and nutrition.^44^ As shown in Fig. 7, four scaffolds (PMMA, PMMA–5% PHP, PMMA–10% PHP, and PMMA–15% PHP) showed different behavior when immersed in deionized water as a function of time. The swelling ratio of the scaffolds increases significantly by adding the PHP complex to PMMA compared with that of PMMA NFs alone. This is explained by the fact that PMMA possesses hydrophobic characteristics; however, the PHP complex has good water-solubility; thus, the addition of the complex increased the degree of swelling of the mats. Notably, PMMA–5% PHP showed a higher degree of swelling ratio with approximately ∼4450% after four days of swelling, compared to that of PMMA, which exhibited a swelling ratio at ∼2100% after one day. Also, PMMA–10% PHP and PMMA–15% PHP represented a high degree of swelling of ∼3700% and 3000% after 2.5 days, respectively. It was observed that a low concentration of the PHP complex (5% and 10%) offered a higher degree of swelling than the PHP concentration of (15%), which was ascribed to the low PHP concentration (5%) being better dispersed and becoming entirely homogenous in the PMMA nanofibers.

### 3.6. Mechanical stability of the PMMA–PHP nanofiber scaffolds

**Fig. 8:**
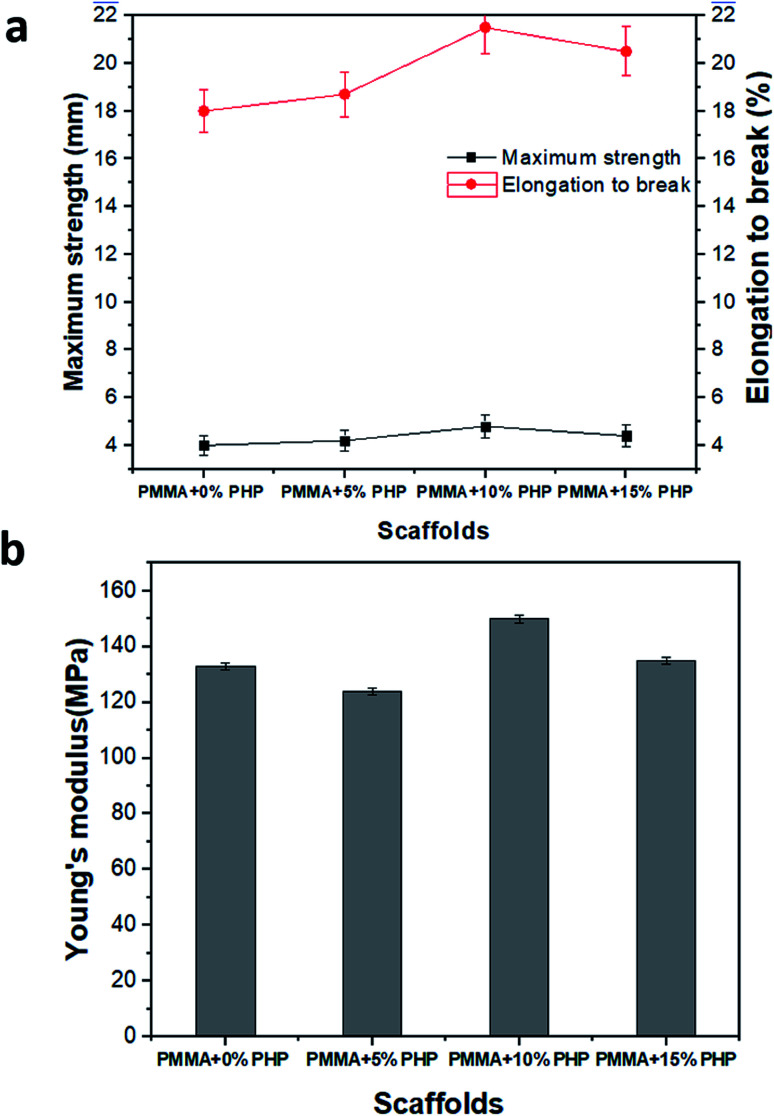
Mechanical properties as (a) maximum strength, elongation-to-break and (b) Young's modulus of (PMMA–0% PHP, PMMA–5% PHP, PMMA–10% PHP, PMMA–15% PHP) nanofiber scaffolds, (mean ± sd, *n* = 3, *p* < 0.05).

Mechanical stability measurements, for example, Young's modulus, elongation-at-break (%), and maximum strength of the nanofibrous scaffolds (PMMA, PMMA–5% PHP, PMMA–10% PHP, and PMMA–15% PHP), were undertaken to estimate the nanofibers' stability under mechanical loads, as displayed in Fig. 8. Interestingly, incorporation of ≥5% PHP complex into the PMMA nanofibers greatly improved the mechanical strength compared to the nanofibers without PHP (*i.e.*, PMMA) nanofibers (Fig. 8a). As previously discussed in the swelling results (Fig. 7), where the incorporation of 10% PHP enhanced the mechanical properties (Young's modulus) of the nanofibers to the highest value in the case of the PMMA + 10% PHP NFs. This indicates that the addition of PHP improves the mechanical stability of the nanofiber; thus, this concentration gave a lower value of swelling than that of the PMMA + 5% PHP NFs. Generally, Young's modulus values of the nanofibers enhanced significantly due to PHP complex incorporation. These results indicate that the compatibility between PMMA and PHP might be realized as a result of the blending mechanism. Meanwhile, the incorporation of PHP into the PMMA solution might create a clear porous interconnected scaffold, which improves the mechanical stability of the nanofibrous mats and in turn facilitates cell adhesion, proliferation, and differentiation.

### 3.7. Hydrolytic degradation of the PMMA–PHP nanofibers

**Fig. 9:**
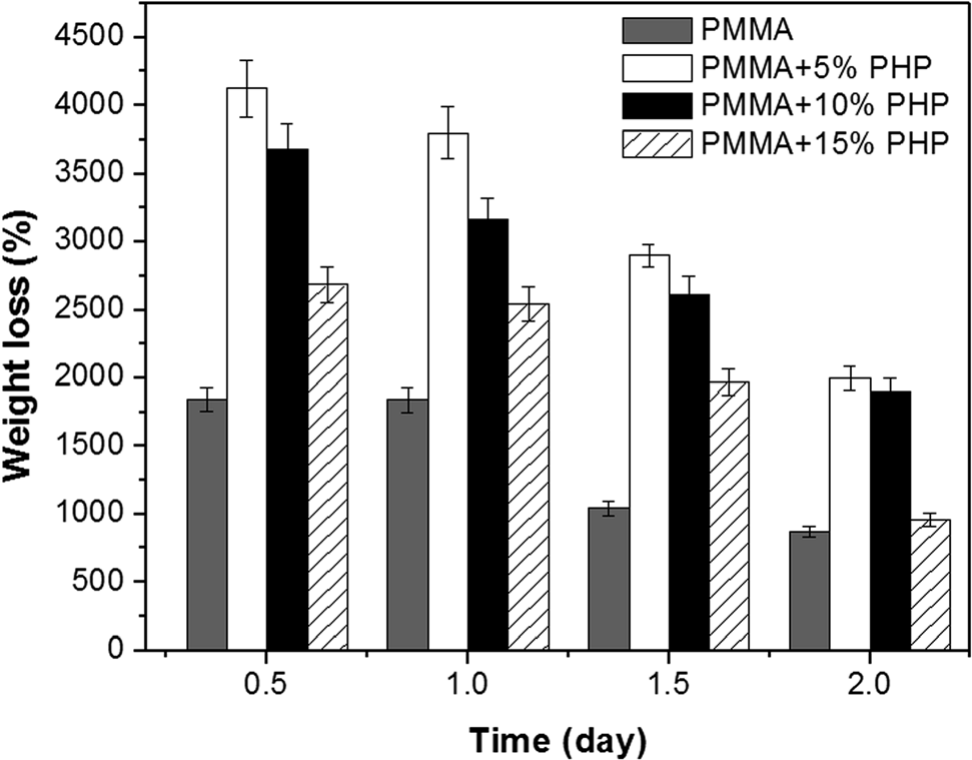
Percentage of weight loss from PMMA–0% PHP, PMMA–5% PHP, and PMMA–10% PHP and PMMA–15% PHP nanofibrous scaffolds, (mean ± sd, *n* = 3, *p* < 0.05).

Fig. 9 represents weight loss (%) over time of the (PMMA, PMMA–5% PHP, PMMA–10% PHP, and PMMA–15% PHP) nanofibrous scaffolds in deionized water. The results show that the weight loss (%) of the PMMA–PHP NFs decreases over 2 days, compared to that of PMMA due to the release of PHP, particularly in the first 12 hours. These results are in accordance with those obtained by Carvalho *et al.*,^45^ who demonstrated that PMMA is a water-insoluble polymer and its degradation could be increased by the addition of different ratios of PEO (10, 20, and 30%) owing to its high solubility in water. The current findings proved that the degradation of the PMMA scaffold was affected the composition of different concentrations of the PHP complex. As mentioned before, PMMA loaded 5% PHP and 10% PHP scaffolds provided a high degree of swelling due to the good dispersion and homogeneity of the PHP complex throughout the nanofibers' interior structure. In addition, PMMA loaded 5% and 10% PHP scaffolds revealed a higher degree of weight loss after two days.

### 3.8. Dissolved oxygen (DO) determination

**Fig. 10:**
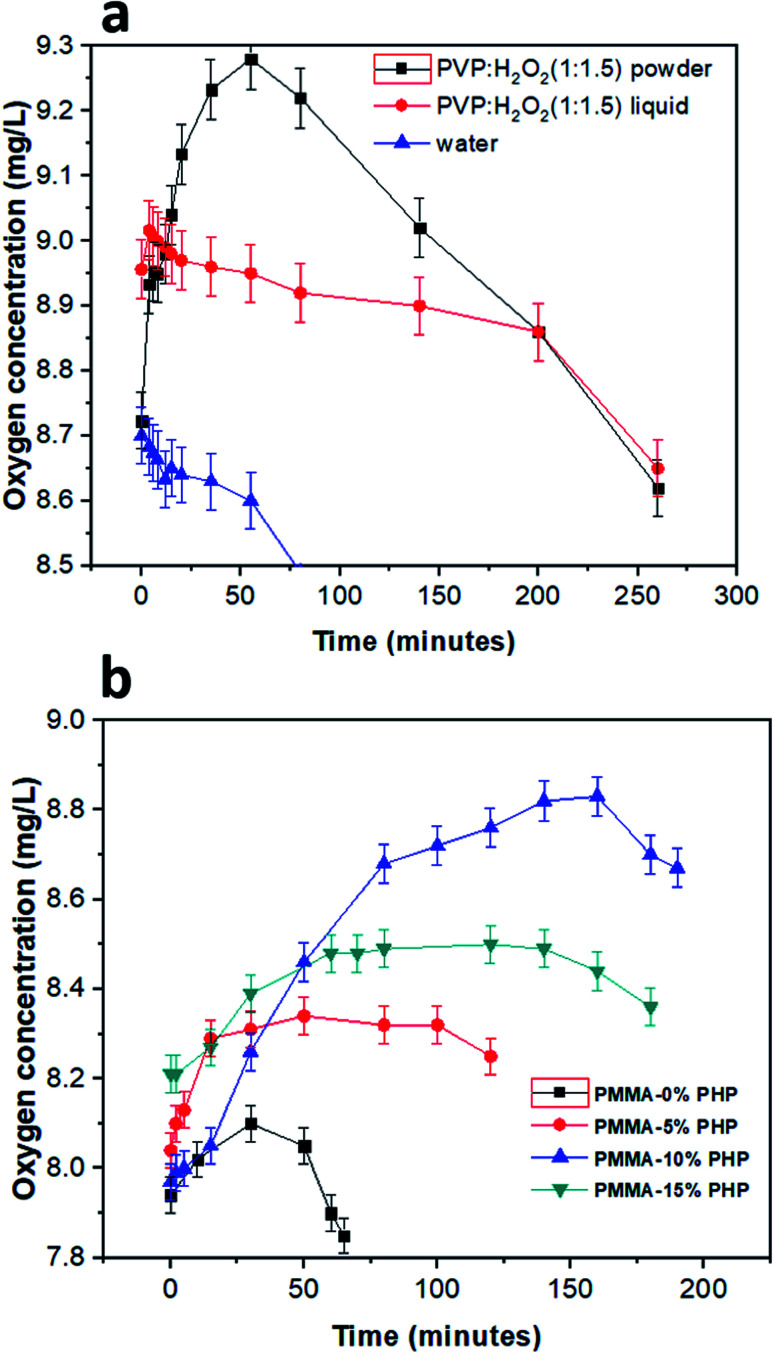
DO concentration (mg mL^−1^) in de-ionized water as function of different nanofibrous scaffolds of (a) (PMMA–0% PHP, PMMA–5% PHP, PMMA–10% PHP, and PMMA–15% PHP), as well as for (b) PHP complex as powder and liquid forms, (mean ± sd, *n* = 3, *p* < 0.05).

The concentration of oxygen in solution with different PHP loaded-PMMA scaffolds is presented in Fig. 10. DO was detected for the PHP complex in two cases as the liquid phase before lyophilizing and in one case after solidification. As shown, the powder PHP complex released oxygen at higher concentrations than in the liquid phase; this result could be explained due to the high content of water in the case of the liquid phase PHP complex and fast decomposition of H_2_O_2_ into H_2_O and O_2_ (Fig. 10a). However, the preparation of the PHP complex as a powder by lyophilization leads to the increased stability of hydrogen peroxide and the removal of excess water. On the other hand, DO concentration was measured for the PMMA scaffolds loaded-PHP complex with different concentrations (5, 10, and 15%), as presented in Fig. 10b, and revealed the amount of released oxygen. The PMMA scaffold loaded 10% PHP complex released the highest concentration of dissolved oxygen (∼8.9 mg L^−1^ after 2.5 h) due to its good dispersion and homogeneity, as was proved before by the results of swelling and degradation properties (Fig. 7 and 9), respectively. The concentration of DO released from the loaded scaffolds compared to the unloaded PMMA scaffold recorded very low change in the DO concentration in the first 20 min due to their porosity. It was expected that the more loaded the PHP complex, the more the oxygen released but the results proved that the PMMA scaffold with 15% PHP complex released oxygen with concentration (∼8.5 mg L^−1^ after 2.5 h) less than that of PMMA–10% PHP; this can be clarified due to the weak dispersion of the PHP complex when the concentration increased by 10% in the scaffold. These results are consistent with the previous results of Ahmed *et al.*,^35^ who revealed the measurement of dissolved oxygen in deionized water released from oxygen nano-bubbles (ONB) compared to air nano-bubbles (ANB). The results represented that DO increases with time intervals (throughout 120 min) in the case of ONB, while DO declines when ANB is dispersed into deionized water.

### 3.9. Cytotoxicity test of the PHP complex and the PMMA–PHP nanofibers

**Fig. 11:**
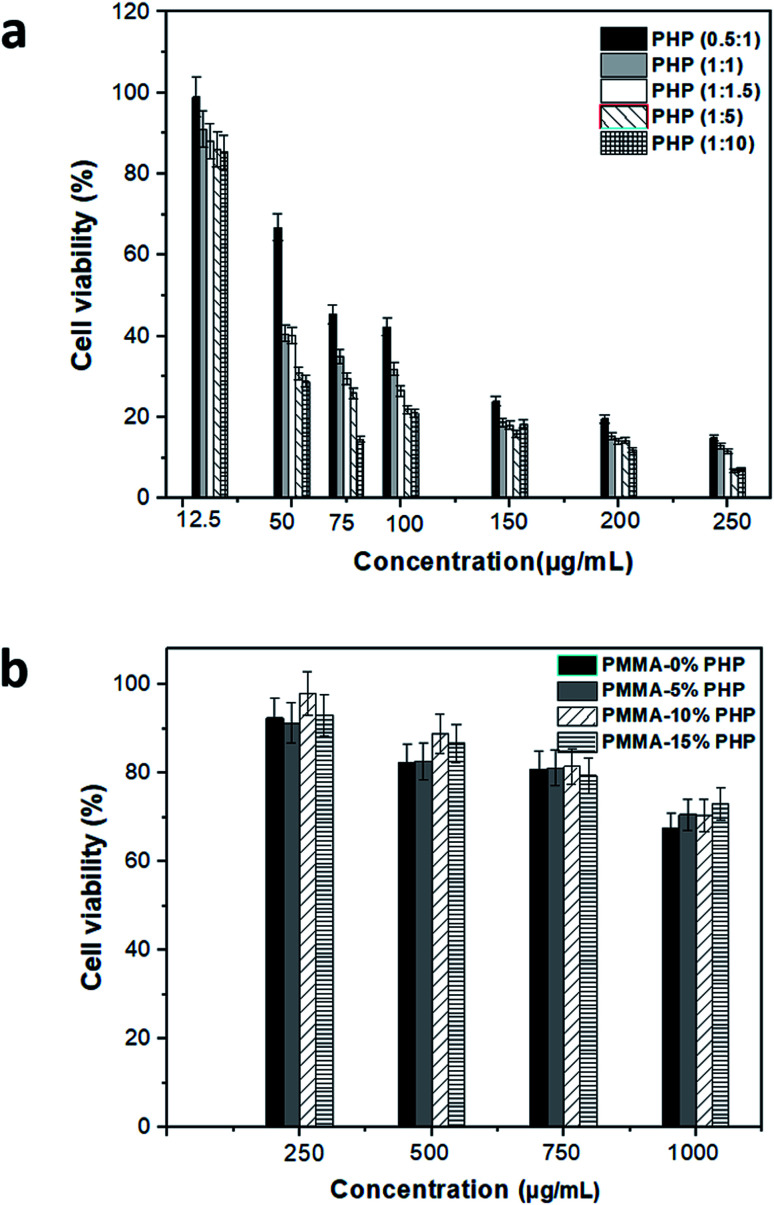
Cytotoxicity assessment of (a) free PHP with different ratio and (b) PMMA loaded with PHP complex (PMMA–0% PHP, PMMA–5% PHP, PMMA–10% PHP and PMMA–15% PHP), (mean ± sd, *n* = 3, *p* < 0.05).

Vero cell viability was determined using the MTT assay to assess the enhancement effect of the PHP complex when loaded into the PMMA scaffolds with different concentrations, as shown in Fig. 11. Different ratios of the PHP complex (*i.e.*, PVP : H_2_O_2_) showed a clear toxic effect on the Vero cells, when treated with high concentration of PHP, as displayed in Fig. 11a. The cell number reduced to the lowest value of *ca.* 15%, when cells were treated with 250 μg mL^−1^; this was recorded for all the ratios of the PHP complex. However, the cell viability was observed to be more than 80%, when it was preserved with a low concentration of the complex of about 12.5 μg mL^−1^. This could be explained as a rapid release of a high amount of H_2_O_2_ occurred as the complex is extensively water soluble and H_2_O_2_-induced cell apoptosis and cell damage. This result is consistent with the results of Yang *et al.*,^46^ who reported the effect of loliolide, which was extracted from *Sargassum ringgoldianum* subsp., to protect Vero cells from the damaging effect of H_2_O_2_. In addition, they proved that the cell viability reduced to 44% when treated with H_2_O_2_ compared to the control cell without any treatment (*i.e.*, 100%); when the cells were cured with 500 μg mL^−1^ of loliolide, the cell viability increased to 60%. On the other hand, the Vero cells presented high viability when treated with different concentrations of the PHP complex loaded into the PMMA scaffolds, as revealed in Fig. 11b. The concentration of 10% PHP loaded into the PMMA nanofibers represented the highest cell viability even when the cells were treated with high concentration of the nanofibers. The cell viability was found to be 92, 91, 98, and 93% when treated with 250 μg mL^−1^ of PMMA + 0% PHP, PMMA + 5% PHP, PMMA + 10% PHP, and PMMA + 15% PHP nanofibrous scaffolds, respectively. As revealed in Fig. 11b, almost all the concentrations of the PMMA + 10% PHP scaffold used to treat the Vero cells showed the highest amount of cell viability of about 75%. It was clearly observed that the toxicity of the PHP complex was extensively reduced when it was blended into the hydrophobic polymer, *e.g.*, PMMA, as the cell viability was 15% when treated with 250 μg mL^−1^ of PHP (1 : 1.5); however, it increased by almost seven times to ∼98% when the cells were incubated with the same concentration (250 μg mL^−1^) of the PMMA + 10% PHP scaffold. This result could be explained as the hydrophilic complex offered a sustained release profile from the PMMA scaffold; thus, the cells possess the ability to convert H_2_O_2_ into water and oxygen, which are safe for the cell.^47^

### 3.10. Anticancer activity of the PHP complex and the nanofibers

**Fig. 12:**
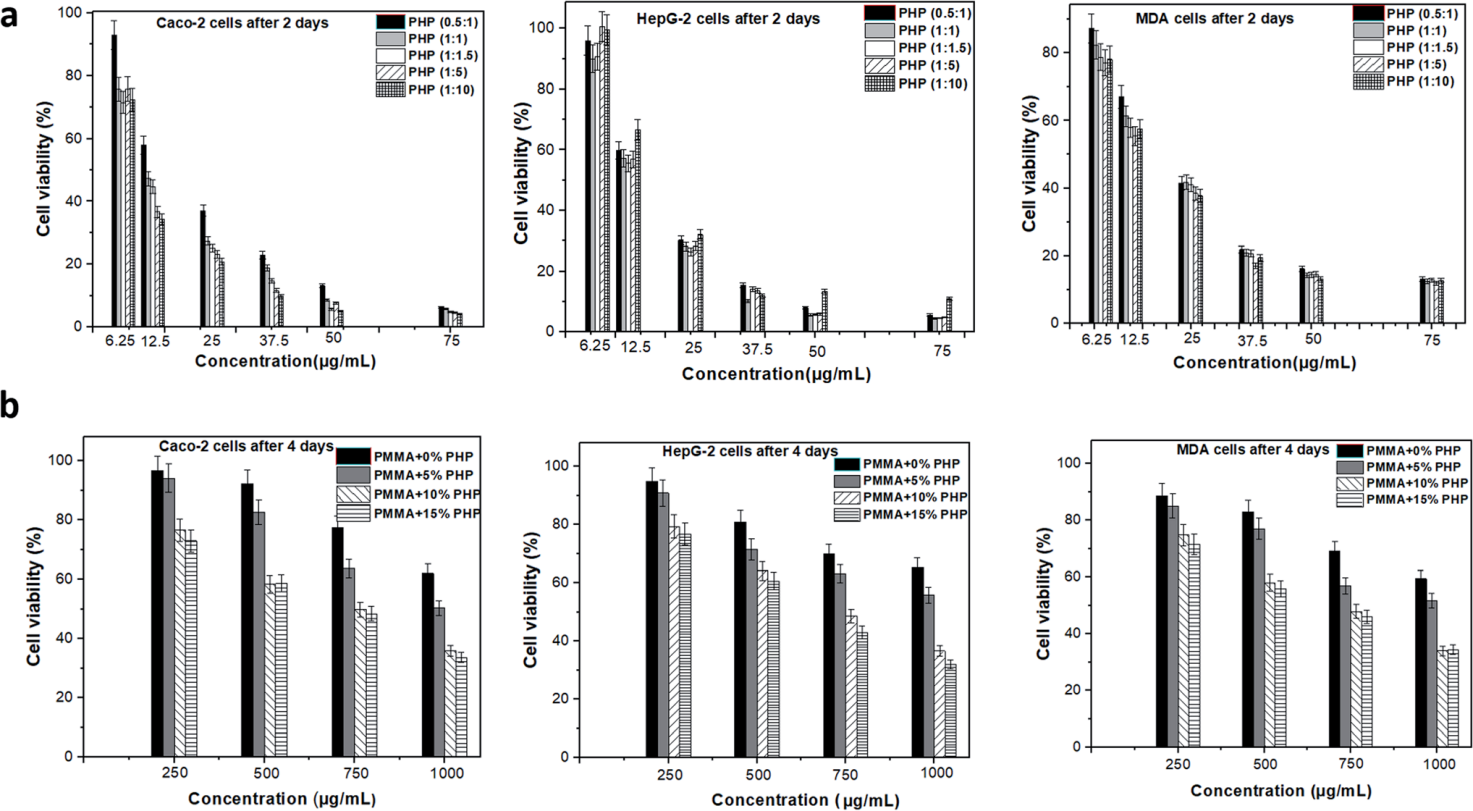
Anticancer assay of different ratios of PHP complex treated with various cancer cell lines Caco-2 cells, MDA cell and HepG-2 cells (upper rows), and with different blends of PMMA–PHP nanofibrous scaffold mats on the same cell lines (down rows), (mean ± sd, *n* = 3, *p* < 0.05).

The anticancer effect of different PHP complex ratios and nanofibrous scaffolds (PMMA + 0% PHP, PMMA + 5% PHP, PMMA + 10% PHP, PMMA + 15% PHP) was studied on different cancerous cell lines (Caco-2 cells, HepG-2 cells, MDA cell), which were specified for colon cancer, liver cancer, and breast cancer, respectively. The IC^50^ values of PMMA–PHP nanofibers and PHP complex powder were estimated using Vero, Caco, MDA, HepG2 cell lines, as shown in Table 2. The viability of different cancerous cells was observed after two days of incubation with the PHP complex as a powder and 4 days of incubation with nanofibers using the MTT assay, as explained in Fig. 12a and b, respectively. The results indicate that all the ratios of the PHP complex have an extensive anticancer effect on various types of cancer cell lines, where the cell viability reduced to 4% when treated with 75 μg mL^−1^ of the PHP complex, as shown in Fig. 12a. However, it was observed previously in the cytotoxicity study that the same concentration has a harmful effect on the normal cell where the Vero cell viability was reduced to 12%. On the other hand, the PHP complex loaded onto the PMMA nanofibers has an obvious anticancer effect on different cancerous cell lines, as clearly shown in Fig. 12b. Both the nanofibrous scaffolds composed of PMMA + 10% PHP and PMMA + 15% PHP represented the lowest viability of all the cancerous cell lines and this was explained by both the nanofiber sheets showing the largest contents of the PHP complex as a source of oxygen release. As revealed in Fig. 12, when cells were treated with one mg mL^−1^ of PMMA + 10% PHP, the concentration of different cancerous cells Caco-2 cells, HepG-2 cells, and MDA cell reduced to 35, 36, and 34%, respectively. Likewise, the viability of the cancerous cells was reduced to 33, 32, and 34% when treated with PMMA + 15% PHP nanofibrous scaffolds. In contrast, in case of the pure PMMA scaffold and the PMMA scaffold-loaded with low concentration of PHP, *e.g.*, PMMA + 5% PHP, showed a low effect on the cancerous cells, where the cell viability reached 65 and 52%, respectively, for almost all the cell lines. The major anticancer effect of the nanofibrous scaffolds was due to the presence of the PHP complex, which released oxygen as an inhibitor for cancer cell growth; this observation was supported with previous observations by Yttersian *et al.*,^48^ who demonstrated that after treatment with hyperbaric oxygen, there was considerable tumor growth suppression especially in both the BT-474 breast cancer cell and the human MDA-MB-231 cell (Table 3).

**Table 3:** Selective index (SI) of the PMMA nanofibrous scaffolds and the PHP complex with different ratios^a^

Sample	Selective index (SI)		
	Caco-2 cells	MDA cells	HepG-2 cells
***PMMA–0% PHP (control)***	***1.7***	***1.6***	***1.5***
***PMMA–5% PHP***	***2.7***	***2.8***	***2.6***
***PMMA–10% PHP***	***4.04***	***4.2***	***3.6***
***PMMA–15% PHP***	***4.2***	***4.7***	***3.7***
PHP (0.5 : 1)	4.3	3.9	4.6
PHP (1 : 1)	3.1	1.6	2.5
PHP (1 : 1.5)	2.2	2.4	1.9
PHP (1 : 5)	3.2	2.1	2.2
PHP (1 : 10)	2.9	1.8	1.9
PVP (control)	1.3	1.7	1.1

a Cells in bold italic mean nanofibers with the PHP complex.

### 3.11. Antimicrobial activity of the PHP complex and nanofibers

The prepared nanofibers composed of (PMMA, PMMA–5% PHP, PMMA–10% PHP, and PMMA–15% PHP) were tested for their antimicrobial activity against yeast, *e.g.*, *Candida albicans*, Gram negative bacteria, *e.g.*, *Escherichia coli* and *Klebsiella pneumoniae*, and Gram positive bacteria, *e.g.*, *Bacillus cereus*. After the mentioned incubation period, no clear zones were noticed from all the tested nanofiber discs, which indicate that they do not have a detectable antimicrobial activity against the tested microorganisms. As discussed from the methodology section, the tested nanofibrous scaffolds were fabricated from a synthetic polymer (*i.e.*, PMMA) dissolved in acetone, which has no antimicrobial activity, and these results are consistent with the published results of Marrez *et al.*^49^ and Sodagar *et al.*,^50^ who confirmed that PMMA dissolved in acetone as a biomaterial does not have antimicrobial activity.

### 3.12. Hemolysis assay of the PHP complex and the nanofibers

The blood hemolysis test of the fabricated NFs was estimated against human healthy peripheral blood. As shown in Table 4, the hemolytic percentage was varied among the tested samples compared to the positive control. Both PMMA–10% PHP and PMMA–15% PHP revealed the lowest recorded hemolytic percentage of 50 and 45%, respectively, compared to the positive control. Interestingly, PMMA and PMMA–5% PHP showed the highest percentage of hemocompatibility of 95 and 77%, respectively. It was clearly observed that the ascending order of the hemolytic percentage of the tested nanofibers against blood RBCs was PMMA–15% PHP < PMMA–10% PHP < PMMA–5% PHP < PMMA. Accordingly, the addition of different concentrations of the PHP complex in hydrophobic synthetic compounds such as PMMA significantly improved the blood compatibility and haemostatic performances of NFs as promising biomaterials.

**Table 4:** Blood hemolysis assay (%) results of PMMA, PMMA–5% PHP, PMMA–10% PHP, and PMMA–15% PHP against human healthy blood

Nanofiber	Hemolysis (%)
PMMA–0% PHP	95
PMMA–5% PHP	77
PMMA–10% PHP	50
PMMA–15% PHP	45
Positive control	100

## 4 Conclusions

In conclusion, the present study explored he fabrication of the PMMA–PHP complex nanofibrous scaffolds as a novel model of biomaterials possessing anticancer properties. The PHP (PVP : H_2_O_2_) complex was used as the source of oxygen, which previously confirmed the role of oxygen in the cancer treatment. The most sustained amount of released oxygen from the PMMA + 10% PHP scaffold was described to have a good dispersion of the PHP complex and showed the highest mechanical properties with smooth nanofibers. Based on the dose manner, the dose (1 mg mL^−1^) of the nanofibers showed an intensive reduction in different cancer cell viability, whereas the cell viability reduced to 30%; however, the same dose revealed highly safe behavior on the normal cell. It was clearly noticed that the PHP complex as a powder has high toxicity even at low concentrations on both normal and cancerous cells; however, the PHP complex toxicity decreased by loading onto hydrophobic nanofibers for avoiding the burst-release of H_2_O_2_. It was observed that the selectivity of the cancer cell increased by the addition of high concentrations of the PHP complex, whereas PMMA + 10% PHP and PMMA + 15% PHP are extremely selective to cancerous cells (Caco-2, MDA-MB-231, and HepG2). According to our findings, the PMMA–PHP complex nanofibrous scaffolds were recommended as possible biomaterials for cancer treatment.

## Funding

No fund was received for conducting this work.

## Ethical statement

All experiments were performed in accordance with the Guidelines of World Medical Association Declaration of Helsinki: Ethical Principles for Medical Research Involving Human Subjects, and approved by the ethics committee at The British University in Egypt (BUE).

## Author contributions

Samar Salim: study design, conceptualization, methodology, formal analysis, investigation, data curation, and writing – original draft; Elbadawy Kamoun: study design, conceptualization, validation, resources, writing – review & editing draft, data curation and supervision; Taher Salah Eldin: review & editing, and supervision; Tarek Taha: methodology; Esmail El-Fakharany: methodology, formal analysis and data curation; M. El-Mazar: review & editing, and supervision; A. Abdel-Aziz: review & editing, and supervision; R. Abou-Saleh: visualization and validation; and S. Evans: writing – review & editing, and supervision. All authors approve the publication of the manuscript in the current version.

## Conflicts of interest

There are no conflicts to declare.

## Supplementary Material

RA-011-D1RA02575A-s001
