# Supplementary material for: Novel oxygen-generation from electrospun nanofibrous scaffolds with anticancer properties: synthesis of PMMA-conjugate PVP–H2O2 nanofibers, characterization, and in vitro bio-evaluation tests
Source: RSC Adv. 2021 Jun 4;11(33):19978–91. doi: 10.1039/d1ra02575a (PMC9033669; doi:10.1039/d1ra02575a)
Supplement: RA-011-D1RA02575A-s001 [file RA-011-D1RA02575A-s001.pdf]

Electronic Supplementary Material (ESI) for RSC Advances.
This journal is © The Royal Society of Chemistry 2021

Novel oxygen-generation from electrospun nanofibrous scaffolds with anticancer properties: Synthesis of PMMA-conjugate PVP-H_2O_2 nanofibers, characterization and *In vitro* bio-evaluation tests

Supplementary Data

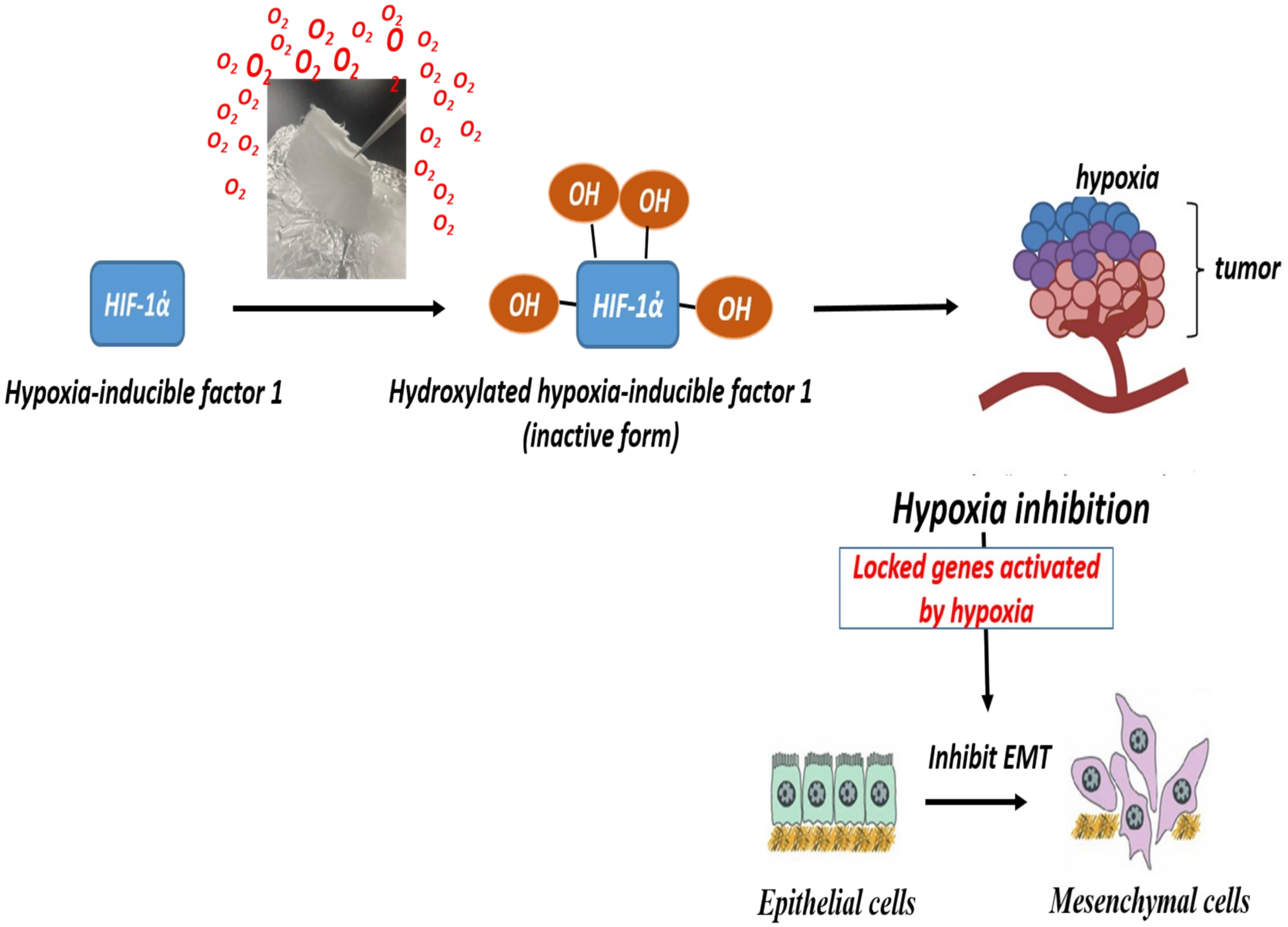

Fig. S1: Schematic diagram representing the proposed mechanism of anticancer effect of released oxygen from nanofiber mats to attack cancer cells.
